# Nanoparticle Recognition on Scanning Probe Microscopy Images Using Computer Vision and Deep Learning

**DOI:** 10.3390/nano10071285

**Published:** 2020-06-30

**Authors:** Alexey G. Okunev, Mikhail Yu. Mashukov, Anna V. Nartova, Andrey V. Matveev

**Affiliations:** 1 Novosibirsk State University Higher College of Informatics, Russkaja Str. 35, 630058 Novosibirsk, Russia; okunev73@mail.ru; 2 Boreskov Institute of Catalysis SB RAS, pr. Acad. Lavrentieva, 5, 630090 Novosibirsk, Russia; avnartova@gmail.com; 3 Scientific-Educational Center “Machine Learning and Big Data Analysis”, Novosibirsk State University, Pirogova Str. 1, 630090 Novosibirsk, Russia; mashukovm@mail.ru

**Keywords:** particle recognition, deep neural networks, scanning tunneling microscopy, particles

## Abstract

Identifying, counting and measuring particles is an important component of many research studies. Images with particles are usually processed by hand using a software ruler. Automated processing, based on conventional image processing methods (edge detection, segmentation, etc.) are not universal, can only be used on good-quality images and need to set a number of parameters empirically. In this paper, we present results from the application of deep learning to automated recognition of metal nanoparticles deposited on highly oriented pyrolytic graphite on images obtained by scanning tunneling microscopy (STM). We used the Cascade Mask-RCNN neural network. Training was performed on a dataset containing 23 STM images with 5157 nanoparticles. Three images containing 695 nanoparticles were used for verification. As a result, the trained neural network recognized nanoparticles in the verification set with 0.93 precision and 0.78 recall. Predicted contour refining with 2D Gaussian function was a proposed option. The accuracies for mean particle size calculated from predicted contours compared with ground truth were in the range of 0.87–0.99. The results were compared with outcomes from other generally available software, based on conventional image processing methods. The advantages of deep learning methods for automatic particle recognition were clearly demonstrated. We developed a free open-access web service “ParticlesNN” based on the trained neural network, which can be used by any researcher in the world.

## 1. Introduction

**Figure 1:**
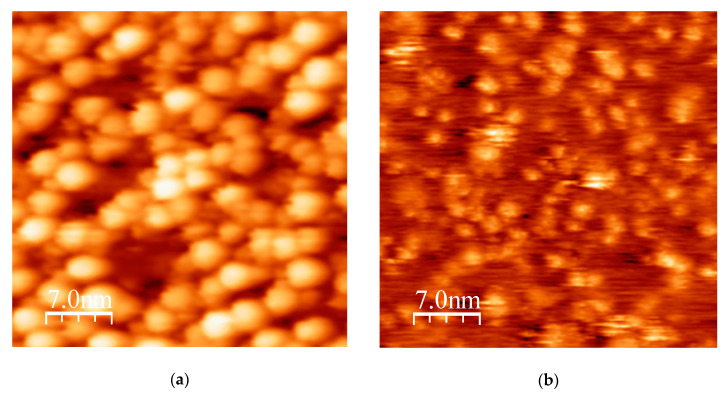
Examples of scanning tunneling microscopy (STM) images of the Pt/highly oriented pyrolytic graphite (HOPG) catalyst in the initial state (**a**) and after treatment in NO_2_ (**b**). Images were recorded in the study, described in [2], unpublished.

In heterogeneous catalysis, the catalyst usually consists of an active component deposited on a support. Active catalyst components are often nanoparticles where the catalytic reaction occurs and reaction products are formed. One of the main characteristics of catalytic activity is the “turnover frequency” of reaction (TOF), defined as the amount of product obtained at one active center per time unit [1]. To calculate TOF, it is necessary to know the particle’s parameters (amount, size, coverage of the surface, etc.). A variety of physical and chemical research methods are currently used to study the characteristics of catalysts e.g., electron microscopy, specific surface analysis, scanning probe microscopy (STM, atomic force microscopy) [1,2]. As a rule, calculating TOF requires processing data on hundreds of particles from several points of the catalyst, the more the better. A similar task arises for researchers in the synthesis and characterization of quantum dots [3,4,5,6]. Software products, widely used in probe methods, including scanning tunneling microscopy, such as WSxM [7], Gwyddion [8], allow the operator to measure particle parameters manually. Their programs implement an automatic selection mode for the image parts with heights above the threshold and show satisfactory results on good-quality images with uniform backgrounds. However, they are unacceptable for noisy pictures or images with an intensity gradient and do not provide a fully automatic mode for recognition of objects and their size determination. Using image preprocessing for stochastic noise elucidation is not always reliable. For example, it is well known that smoothing procedures lead to expanding object boundaries, influencing measured sizes. From this point, median or Fourier filters are better but still can cause artifacts. At the same time, achieving good-quality STM images is not always possible; for example, for in situ STM experiments, for the samples after hard treatments or after chemical reaction (Figure 1) [2]. Complete “cleaning” of such images by processing without deformation is not always implementable too. Moreover, recording a single STM image can take hours as an operator may prefer manual counting instead of rerecording the image to get the desired quality for automatic processing.

Therefore, high-level microscopists still must spend a lot of time tediously counting and manually measuring particle size using a software ruler.

Since 2012, a new approach to image analysis has been actively developing. This method takes into account the context in which objects are located and allows for use of images with marked objects to train the recognition software in automatic mode. It is based on deep convolutional neural networks [9]. The first neural networks were primarily used to classify images. Currently, software products based on deep neural networks can determine the type of object and perform semantic image segmentation, that is, identify pixels belonging to this object [10,11].

In recent years, there have been publications devoted to the use of deep learning for automatic object recognition in materials science and related fields. For example, a number of studies were aimed at searching for defects in metals [12,13,14,15,16] including images of atomically resolved scanning transmission electron microscopy [17], classification of objects in scanning electron microscope images [18], and determining bubbles sizes in thermophysical processes [19].

It is worth noting that in the past few years, decent results have been achieved in the field of transmission electron microscope (TEM) images processing, both using semiautomatic tools and using neural networks. Semiautomatic tools, as a rule, include preprocessing of noisy TEM-images; for example, averaging and background subtracting, level-based segmentation and edge detection [20,21,22]. The main complication of this approach is the mandatory selection of empirical parameters, which leads to a loss of the universality of the approach. In 2019, simultaneously our first work [23], our colleagues applied the MO-CNN neural network [24] and the Mask-RCNN [25] to find the localization of nanoparticles on TEM-images, the size of round particles was finally determined by fitting by circles. Meanwhile, TEM-images analyzed in the cited papers are characterized by uniform and homogenous noise, the particles are clearly visualized and have a rounded shape. Our task of recognizing nanoparticles on STM images is more complicated and provides specific issues.

In this communication in continuation of our first work [23], we describe in detail the procedures of training the Mask-RCNN, fitting particles by 2D Gaussian function and make a comparison with available software that uses conventional semiautomatic tools of particle detection. Besides, we have created and describe a web service “ParticlesNN” for automatic search and recognition of nanoparticles on images of probe microscopies using a neural network trained by us. The web service is available to any researcher from anywhere in the world.

## 2. Materials and Methods

### 2.1. STM Data

A model catalyst consisting of platinum and palladium nanoparticles (99.99%, Alfa Aesar, Ward Hill, MA, USA) deposited on highly oriented pyrolytic graphite (HOPG, SPI Supplies, West Chester, PA, USA) was used in this work. The particles were deposited by thermal vacuum vapor deposition [2].

The STM studies were conducted using a scanning tunneling microscope SPM 100 VT (RHK Technology, Troy, MI, USA). Measurements were taken in the constant-current mode using cut Pt–Ir tips (RHK). The resulting raw STM images were analyzed using XPMPro 2.0 (RHK) and WSxM software packages (Nanotec Electrónica, Madrid, Spain) [7].

For the neural network training and verification we used STM images, which were obtained from the raw data without any further quality improve image processing, such as smoothing, denoising, etc., since: (a) additional processing can distort the image and some information can be lost; (b) correct processing cannot be always possible. Besides, noise and distortions are not crucial for the human eye, which is able to skip them, and thus, the human-like tool based on a neural network also has to be able to process raw data.

### 2.2. Datasets

The training dataset consists of 23 STM images and the corresponding COCO format files [26] with the annotations of 5157 nanoparticles labeled by the operator.

Dataset images were obtained from original STP files, which were saved in an ASCII matrix file format. This file format comprises a header with general information and a height map measured in the experiment. Height at the pixel location *H_pix_* was further converted to the intensity of pixels *I_pix_* of an 8-bit grayscale image:

(1) $$I_{pix}=\frac{H_{pix}-H_{min}}{H_{max}-H_{min}}\cdot 255$$

where *H_max_*, *H_min_* are the maximal and minimal heights recorded in the experiment. The 255 multiplier coefficient was set to match a 256 grayscale format. Finally, images were converted to RGB format by setting the same value of color intensity in all three RGB channels.

Particles were labeled as polygons in the LabelMe program [27] on images colored in a fiery-palette (Julio palette in WSxM). After being unloaded from LabelMe, JSON files with annotations were converted to COCO format, forming files with the annotations of particles.

The test dataset was prepared in the same way. It contains three images with 695 labeled particles and the corresponding COCO format files.

### 2.3. Neural Networks

Neural networks in the Cascade Mask-RCNN family [28] were used with backbones of either X-101-64 × 4d-FPN or HRNetV2p-W32 and pretrained on the COCO dataset [26]. The networks were fine-tuned for 500 epochs with a learning rate of 0.001 in epochs 0–99; 0.0001 in 100–250 epochs and 0.00001 for the following epochs. Both the original height maps 512 × 512 resolution and resized copies (2× and 3×; the 4× rescaled image failed to fit a single GPU memory) of the images were used for training by adjusting the img_scale parameter of the config file to the required image size.

### 2.4. Evaluation

The mean averaged precision mAP [29] with a set of threshold values (0.5, 0.55, 0.6, 0.65, 0.7, 0.75, 0.8, 0.85, 0.9 and 0.95) was used as a quality metric for predicted nanoparticles. In brief, metrics estimate the extent of the intersection of union between prediction and ground truth averaged over all classes, and the set of threshold values. Values of mAP of 0 (or 0%) correspond to poor predictions while mAP close to 1 (or 100%) signals pixel-to-pixel coincidence of predictions and ground truth. mAP metrics were evaluated using a COCO API tool [30] with annotations (annType) of the “bbox” type and a maximum particle number (maxDets) of 500.

Training and recognition were carried out on the HPE Apollo 6500 Gen10 graphics server with eight NVIDIA Tesla V-100 graphics accelerators at the Novosibirsk State University Higher College of Informatics.

### 2.5. Postprocessing

While neural networks produce predictions of particle contours, it is important to have a well-defined, clear algorithm to refine contours of predicted particles that uses a conventional approach for particle contour determination. In this work, we used a 2D Gaussian fit to approximate particle shape. This is a widely used approach for size evaluation [31,32,33,34].

**Figure 2:**
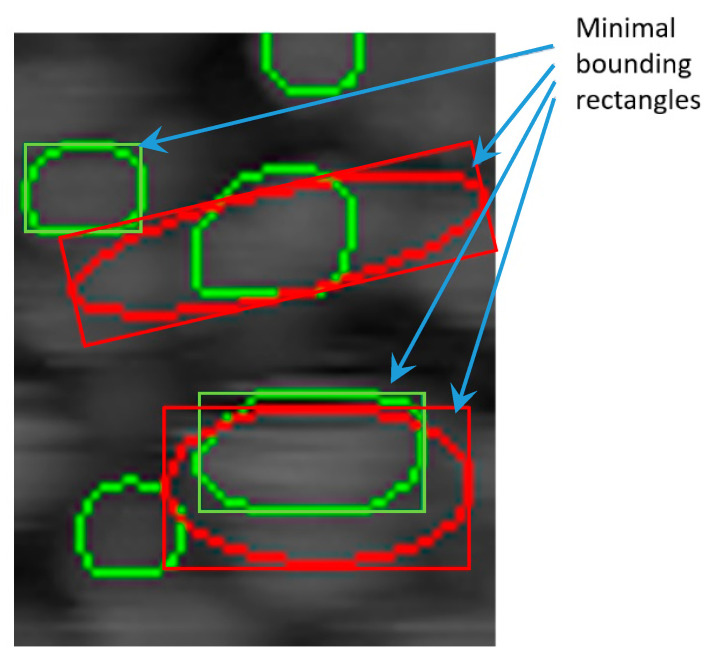
Illustration of the predicted contours refining. Predicted contours are shown in green, refined are in red and minimal area bounding rectangles are the same color as the corresponding contours.

We realized postprocessing procedure, which consists of fitting the particles with a 2D Gaussian surface with seven parameters:

(2) $$H\left(x,y\right)=H_{0}+H_{m}\cdot exp\left(-\frac{{\left(x-x_{0}\right)}^{2}}{s_{x}^{2}}-\frac{{\left(y-y_{0}\right)}^{2}}{s_{y}^{2}}-\frac{\left(x-x_{0}\right)\left(y-y_{0}\right)}{s_{xy}^{2}}\right)$$

where $H_{0}$ is the offset of the surface, $H_{m}$ is the amplitude of the Gaussian surface, *x_0_*, *y_0_* are the coordinates of the surface maximum and *s_x_*, *s_xy_*, *s_y_* are the dispersions. Predicted masks were taken as an initial guess on the location of particles. For each mask, an outer contour was evaluated. Effective particle size *d_eff_* was calculated as a diameter of the circle of the same area as the area of the mask. The entire image was then Gaussian smoothed with a *d_eff_*/4 size kernel. Then, the predicted contour was doubled in linear size while its center of mass was still in the same position. Finally, for close particles, the fit area was refined to exclude intersecting parts as shown in Figure 2. The height map inside the extended contour was approximated with a 2D Gaussian function to obtain fit parameters. Finally, half height was calculated as:

(3) $$H_{min}=max\left(H_{0},\;H_{\min\_pred}\right)$$

(4) $$H_{max}=min\left(H_{\max\_fit},\;H_{\max\_pred}\right)$$

(5) $$H_{half}=\frac{H_{min}+H_{max}}{2}$$

where *H_min_pred_*, *H_max_pred_* are the minimal and maximal experimental heights inside the extended contour after smoothing, and *H_0_*, *H_max_fit_* are the parameters in the Gaussian fit equation and the value of the Gaussian fit function at x_0_, y_0_, respectively. The cross-section of the horizontal plane with Gaussian fit at the half height was considered as the refined border of the particle.

Finally, the width and height of each particle was calculated as the length and width of minimal bounding rectangles for the predicted contour and for the refined contour.

## 3. Results

### 3.1. Training on a ”Rough” Dataset

**Figure 3:**
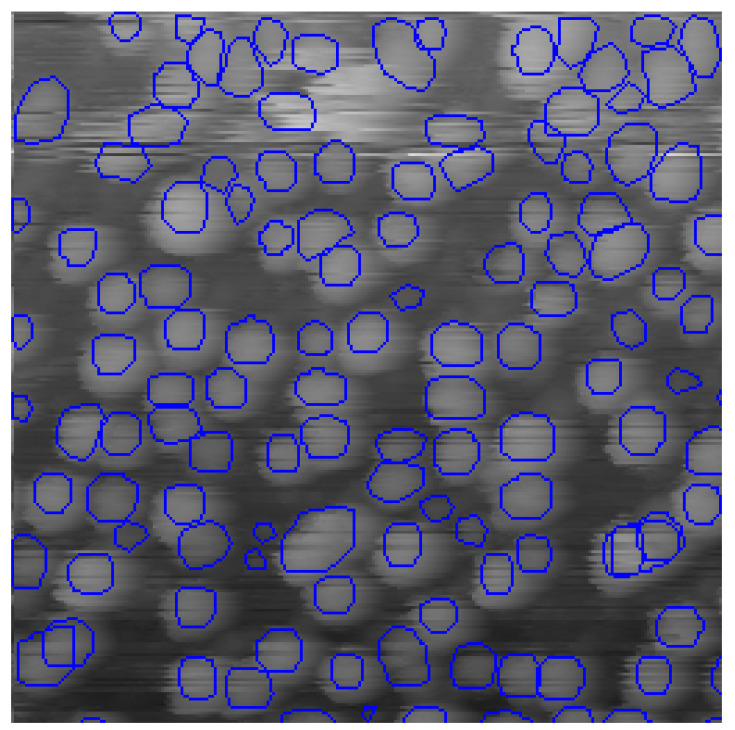
Crop of the STM image labeled without paying attention to actual particle borders, the “rough” dataset. Ground truth labels are shown as blue contours.

The goal of this research was to obtain a credible automatic statistical analysis of STM data. The required data include particle concentration on the surface and particle size distribution. Our first approach was to find particles using a convolutional neural network, then refine the particle borders using a predetermined algorithm. To accomplish this, the training data set was labeled without paying much attention to the actual particle size and greater attention to the total number of particles. We refer to this dataset as “rough”. The crop of labeled data is depicted in Figure 3. The “rough” training dataset comprises 15 images with 3791 particles.

Our fitting results were very sensitive to the size of STM images used during training (Table 1), Nos. 1–3. To a lesser extent, results depend on the backbone type, Nos. 3 and 5. A Cascade Mask-RCNN powered by an X-101-64 × 4d-FPN backbone and trained at three-times rescaled images, No. 3, demonstrated the best particle count accuracy, determined as the ratio of correct predictions to the total number of ground truth contours. It should be noted that the 1536 × 1536 image size is close to the limit of the NVIDIA Tesla V-100 state-of-the-art server graphic accelerator. The four-times rescaled image failed to fit a single GPU memory. The poor mAP metrics can be apparently explained by the low intersection of predicted contours with ground truth contours because of the training on the “rough” dataset.

**Figure 4:**
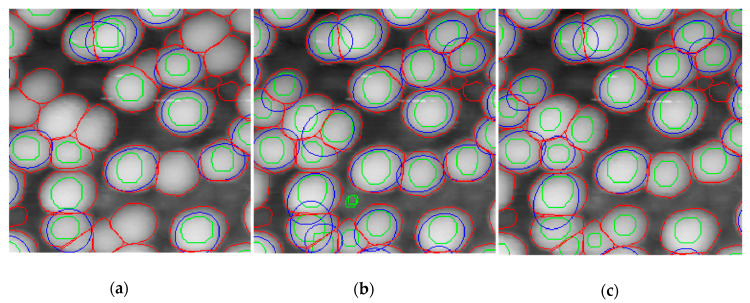
The results of particle recognition on an image from the test dataset by X-101-64 × 4d-FPN backboned Cascade Mask-RCNN network trained on 1-, 2- and 3-times rescaled training images of the “rough” dataset, (**a**), (**b**) and (**c**) correspondingly. Ground truth contours are red, predicted contours are green and Gaussian fitted contours are blue.

Figure 4 illustrates the difference in particle recognition by the neural network, trained on different dataset image sizes. While the image count quality improves with the size of the training image, the quality (mAP) of the contours prediction by the neural network remains poor. Fitting the predicted particle profiles with a Gaussian surface improves the situation to some extent. Gaussian refined contours follow ground truth much better than the raw predicted contours. However, in almost half of the cases, the Gaussian fitting failed to refine particle contours. On the test dataset, only 353 contours were successfully refined of 624 predicted, when the total amount of ground truth particles in the dataset was 695. Thus, almost half of the particles were outside statistical measurement which is unacceptable for credible measurements of size distribution. We consider the particle clumping as the main obstacle to a Gaussian fitting.

Thus, a neural network trained on a “rough” dataset shows the high accuracy of particle recognition. Postprocessing by Gaussian fitting refined the predicted contours to a high extent. However, if there are clumped particles on the surface, the fitting procedure reduces the number of analyzed particles, which can significantly diminish the statistical plausibility of determining the particle size.

### 3.2. Training on a ”Precise” Dataset

**Figure 5:**
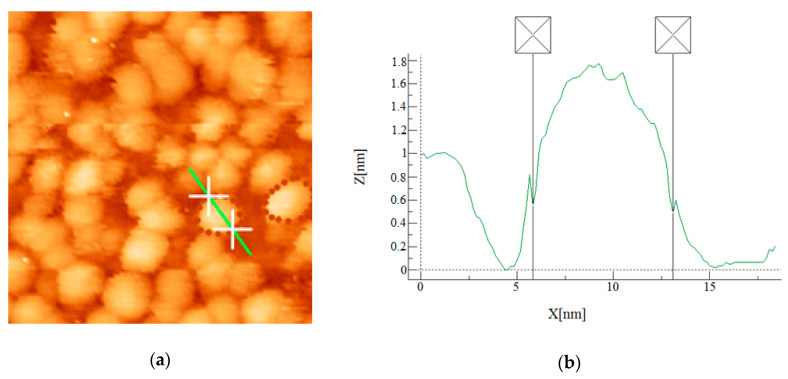
Illustration of the contour quality check. (**a**) Julio color scheme colored STM image with two particles ground truth labeled contours. (**b**) Particle profile, extracted from STM data using WSxM software, showing the position of ground truth labeled contour on the profile of the particle.

Our next approach was to provide a high-quality dataset with credible particle borders. In order to get such a training dataset, eight images containing 1186 particles were carefully labeled by an expert along the visual borders of the particles, forming ground truth contours. The widely used Julio color scheme was applied to diminish labeling errors. Figure 5 shows that such labeling along the visual borders of the particles is reasonable. Meanwhile, an additional check for this labeling technique accuracy was done for one of the test images. The diameters of 39 arbitrarily chosen particles from the image were measured in the WSxM program using manual precise profile analysis. The average diameter was 6.1 nm. The projected diameter (diameter of a circle, which has the same area as the particle) for the same 39 ground truth contours was averaged and it was 5.8 nm. A 5% value of discrepancy for this small subset of the test dataset looks reasonable and can be explained by the ambiguity of manual measurements.

**Figure 6:**
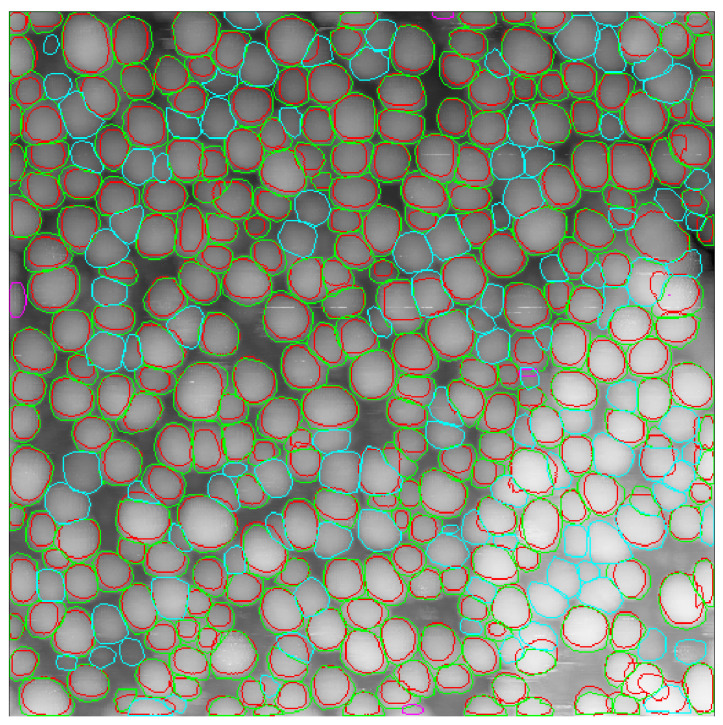
STM image of nanoparticles from the test dataset with 377 ground truth contours and 272 predicted contours. True positive predicted contours are marked red, corresponding ground truth contours are green, false positive ground truth contours are pink and false negative ground truth contours are cyan. Image 1, backbone type X-101-64 × 4d-FPN, scale 3×, “precise” training dataset.

**Figure 7:**
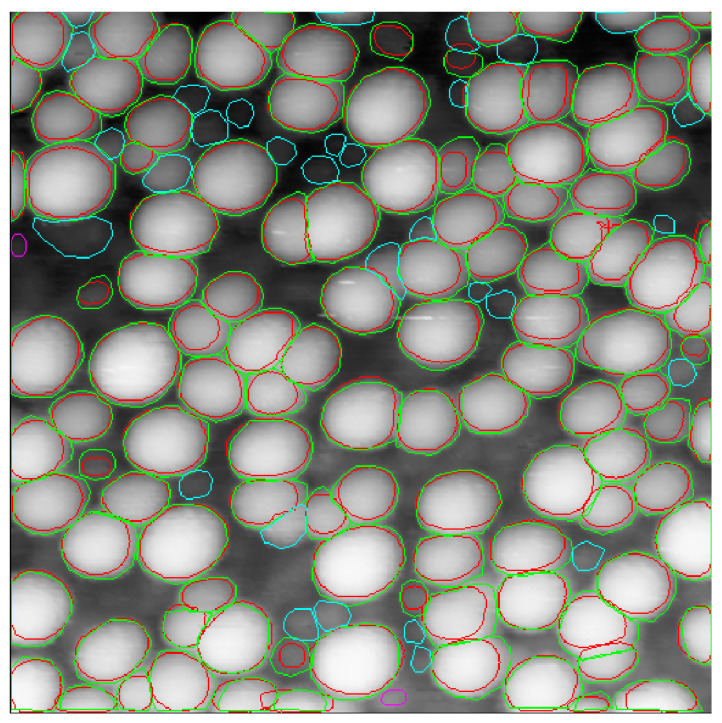
STM image of nanoparticles from the test dataset with 146 ground truth contours and 118 predicted contours. True positive predicted contours are marked red, corresponding ground truth contours are green, false positive ground truth contours are pink and false negative ground truth contours are cyan. Image 2, backbone type X-101-64 × 4d-FPN, scale 3×, “precise” training dataset.

**Figure 8:**
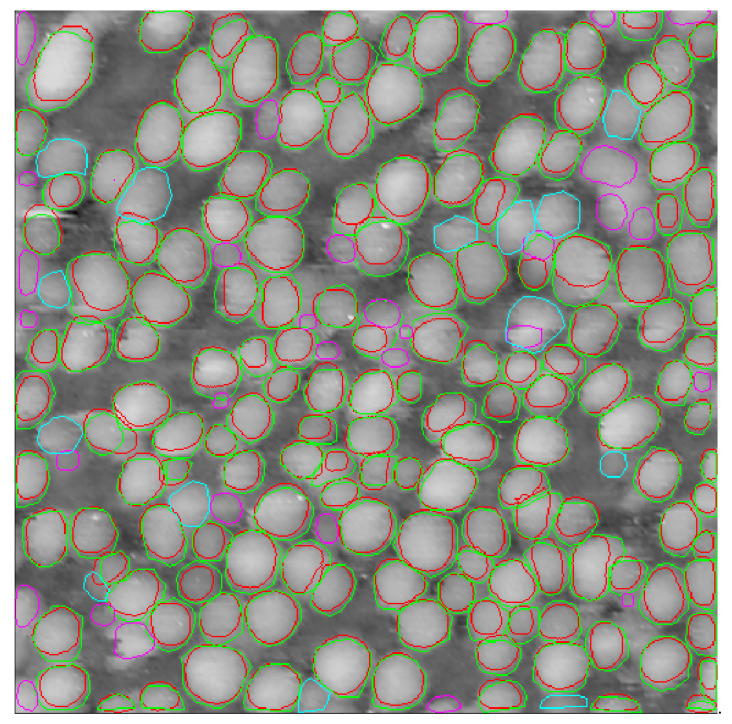
STM image of nanoparticles from the test dataset with 172 ground truth contours and 190 predicted contours. The true positive predicted contours are marked red, corresponding ground truth contours are green, false positive ground truth contours are pink and false negative ground truth contours are cyan. Image 3, backbone type X-101-64 × 4d-FPN, scale 3×, “precise” training dataset.

A Cascade Mask-RCNN network powered by an X-101-64 × 4d-FPN backbone was trained on a” precise” dataset using a 3× rescale of the training images (last line in Table 1). While there was a small loss in the number of correctly determined particles, we observed a huge jump in mAP. Visual inspection reveals a very good correspondence of predicted contours with ground truth counterparts (Figure 6, Figure 7 and Figure 8). We attribute the slightly lower mAP value of 27.9% as compared with state-of-the-art mAP of about 40% for the Cascade Mask-RCNN fitting of the COCO dataset [28] to the small size of the training dataset.

To analyze the quality of neural network prediction, we distribute predicted contours into three general categories:

(a) Predicted and ground truth contours to a large extent coincide, or at least mark the same particle.

(b) Predicted contours to a large extent missed any ground truth contours.

(c) Ground truth contour has no close correspondence to predicted contours.

These cases can be considered as true positive (TP), false positive (FP) and false negative (FN) predictions, respectively. Using this classification, one can calculate precision and recall (accuracy) for each recognized image and the test dataset as a whole:

(6) $$precision=\frac{TP}{TP+FP}\;,\;recall=\frac{TP}{TP+FN}$$

Any predicted contour with a center inside of the ground truth contour was considered a TP prediction. Other predicted contours were FP. Ground truth contours passed by any predicted contours were set as FN. If predicted contour included more than one ground truth contour, only one ground truth contour was set as a TP and all other included contours as FN. Table 2 summarizes the inference results for all three test images with respect to the quality of the particle count.

It was noted that when images had a larger number of particles recognized, the number of particles left unrecognized increased considerably, see Figure 6 and corresponding recall in Table 2. Currently, our practice shows that to improve the accuracy of recognition, one should avoid more than 200 particles on the analyzed image.

### 3.3. Refining Predicted Contours

Table 3 lists numbers of ground truth, predicted and Gaussian fitted contours for each image from the test dataset. Unfortunately, there were a very limited number of cases when the Gaussian surface fit particles. STM particle shapes are far from ideal, including statistical current noise, instrumental artifacts and complications that arise when particles are close to each other.

**Figure 9:**
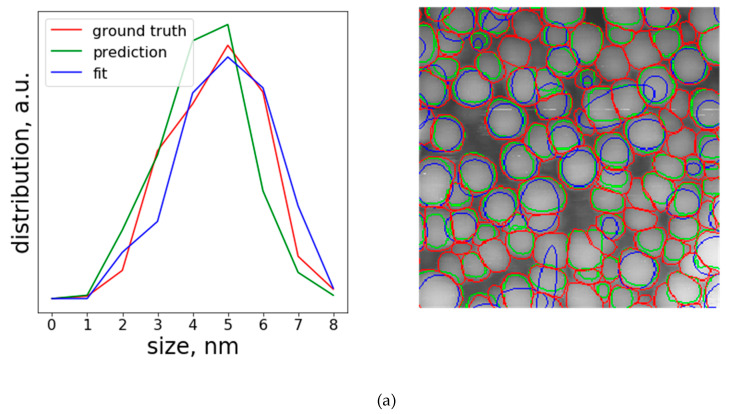

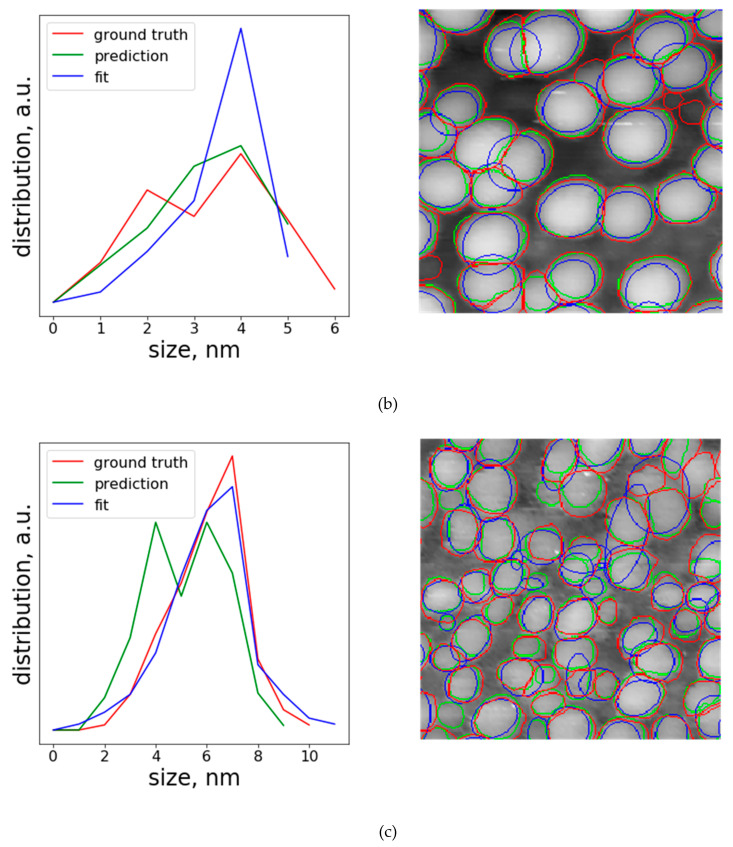
Particle size distributions (left panels) and corresponding particle contours (right panels), Image 1 (**a**), 2 (**b**) and 3 (**c**). For better visualization, crops of the images are shown. The ground truth contours are marked red, predicted contours are green and Gaussian fitted contours are blue.

Different types of contours are visualized in Figure 9. As mentioned previously, the number of refined (blue) contours is much lower than the number of ground truth (red), or predicted (green) contours. On the other hand, histograms calculated for both predicted and Gaussian fitted contours follow ground truth surprisingly well. This is even more surprising after visual inspection of the fitted contours, which are often very far from ground truth labels. However, one can observe the tails in the larger diameter region of the fitted contours histogram. These tails arise from distorted elongated fitted contours that failed to refine particle borders properly. The average projected particle diameters calculated from histograms considered as mean particle sizes confirm this observation (Table 4). Mean particle sizes calculated from Gaussian fitted contours are systematically higher than the ground truth values for the reasons described above.

Thus, we should summarize that our attempt to provide a universal, clear and reliable algorithm for refining particle contours has not been reached. Today, deep neural networks are probably the only solution to obtain nearly human quality image processing.

### 3.4. Comparison with Other Software

It is reasonable to compare the results of particle recognition by the trained neural network with use of software that is not based on deep learning algorithms. Unfortunately, advanced semiautomatic tools, which include preprocessing (averaging and/or background subtracting), level-based segmentation and edge detection [20,22] are not generally available. So, we used the “flooding” procedure in WSxM software products. The procedure highlights areas that are higher than the threshold defined by the operator [7].

**Figure 10:**
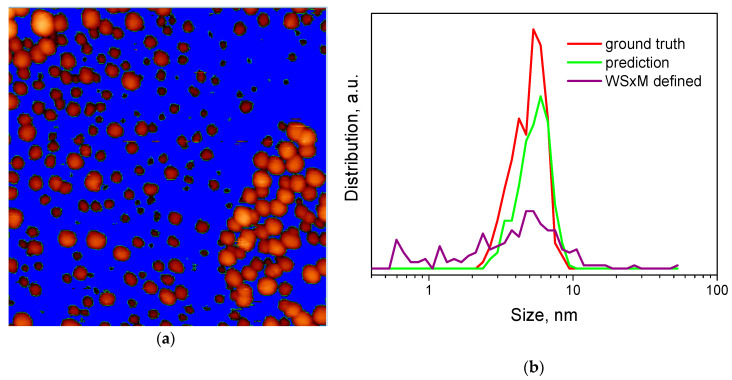
Processing of Image 1: (**a**) Results of the application of the “flooding” procedure in WSxM software products; (**b**) Size distributions of the 196 contours defined by WSxM software (purple), 272 particles recognized in our work (green) as well as 377 contours of ground truth (red), x-scale presented in log10 units.

In Figure 10a the results of the application of the “flooding” procedure in WSxM software products for Image 1 are shown. Some unusual features are clear such as the procedure works poorly for image areas with an intensity gradient and, moreover, the program defines all noises as objects. So with higher intensity islands, the operator has to fix the higher threshold which leads to a decrease in particle size, still leaving islands of increased intensity that are considered by the procedure as a single particle. Finally, the procedure loses a number of particles, reduces the size of the found particles and defines artificial big particles.

Figure 10b shows a comparison of the size distributions of the contours on Image 1 as defined by WSxM software products, by the trained neural network and by the operator (ground truth). In Table 5, the statistical parameters for distributions are shown. It can be seen that “flooding” gives wrong and artificial numbers of particles. The mean particle size for this image happened to be close to the ground truth one, although the standard error of mean is considerably higher. Figure 10b clearly shows the broader histogram for particles, determined by “flooding”. A similar analysis was performed for Images 2 and 3, see Figures S1 and S2 and Tables S1 and S2.

### 3.5. Online and Public Resources

To make it possible for any user, anywhere in the world to use the results of our work on particle recognition on STM images, we have been developing a web service “ParticlesNN” [35], see the short description in the Supplementary Materials and more detailed on the service’s website.

Additionally, on the website of the service, we have released training and test dataset files: initial STM data in TXT format, corresponding BMP images and files with annotations of labeled particles in COCO format.

## 4. Conclusions

In this study, we used deep learning for the automatic recognition of nanoparticles on STM images. The results of the proposed approach were compared with the outcomes of other software products based on conventional image processing methods. The advantages of using deep learning methods for automatic particle recognition were clearly demonstrated. Based on the trained neural network, we developed a web service “ParticlesNN” that has the following features that differ from other software products:

1. It is possible to process images containing noise, artifacts that are typical for probe microscopy images, without additional processing;
2. The user can adjust automatically determined contours with the help of external software products;
3. Joint statistical processing of the image sets is available;
4. Processing results are displayed in the form of a histogram and tables where information on all identified objects is available.

This approach to particle recognition using neural networks allows us to improve the quality of recognition over time by accumulating marked data. To the best of our knowledge, this is the first time that computer vision based on deep learning has been successfully applied to the automated recognition of nanoparticles on STM images. In addition, the web service is able to work with images of any objects represented as intensity spots such as microparticles (obtained for example by SEM), biological cells, etc.

## Figures and Tables

**Table 1:** The results of particle recognition by Cascade Mask-RCNN with various backbone types and the sizes of STM images used during training.

No.	Backbone Type	Image Size (Rescale)	Dataset	Contours	Batch Size	Accuracy	mAP
1	X-101-64 × 4d-FPN	512 × 512 (1 times)	Rough	Predicted	12	0.24	0.000
2	X-101-64 × 4d-FPN	1024 × 1024 (2 times)	Rough	Predicted	4	0.73	0.000
3	X-101-64 × 4d-FPN	1536 × 1536 (3 times)	Rough	Predicted	1	0.83	0.000
4	X-101-64 × 4d-FPN	1536 × 1536 (3 times)	Rough	Fitted	1	0.51	0.000
5	HRNetV2p-W32	1536 × 1536 (3 times)	Rough	Predicted	1	0.82	0.000
6	X-101-64 × 4d-FPN	1536 × 1536 (3 times)	Precise	Predicted	1	0.78	0.279

**Table 2:** Summary on the quality of the particle count in the test dataset.

Image No.	Particle Count			Precision	Recall (Accuracy)
	TP	FP	FN		
1	266	6	111	0.98	0.71
2	115	3	31	0.97	0.79
3	158	32	14	0.83	0.92
Total	539	41	156	0.93	0.78

**Table 3:** Numbers of different types of contours, on the test dataset images.

Backbone Type	Number of Contours Found		
	Ground Truth	Predicted	Gaussian Fitted
1	377	272	172
2	146	118	95
3	172	190	147

**Table 4:** Mean particle sizes calculated from histograms of different types of contours. The accuracies for predicted and Gaussian fitted contours compared with ground truth are shown in parenthesis.

Image No.	Mean Particle Size, nm		
	Ground Truth	Predicted	Gaussian Fitted
1	5.19	4.87 (0.94)	5.38 (0.96)
2	3.82	3.85 (0.99)	4.15 (0.92)
3	5.32	4.62 (0.87)	5.33 (0.99)

**Table 5:** Processing of Image 1: number of particles and their mean particle sizes calculated from histograms of different types of the contours.

Method for Determining Particle Size	Number of Particles	Mean Particle Size, nm	Standard Error of Mean
Procedure “flooding”, WSxM software	196 ^1^	4.93 ^1^	0.33 ^1^
Neural network Cascade Mask-RCNN, used in this work (predicted)	272	4.87	0.07
Ground truth	377	5.19	0.06

^1^ Depends on threshold.

## Supplementary Materials

The following are available online at https://www.mdpi.com/2079-4991/10/7/1285/s1, Figure S1. Processing of Image 2: Results of the application of the “flooding” procedure in WSxM software products; Size distributions of the 69 contours defined by WSxM software, 118 particles recognized in our work as well as 146 contours of ground truth. Table S1. Processing of Image 2: number of particles and their mean particle sizes calculated from histograms of different types of the contours. Figure S2. Processing of Image 3: Results of the application of the “flooding” procedure in WSxM software products; Size distributions of the 115 contours defined by WSxM software, 190 particles recognized in our work as well as 172 contours of ground truth. Table S2. Processing of Image 3: number of particles and their mean particle sizes calculated from histograms of different types of the contours. Web service description.
