# Supplementary material for: Nanoparticle Recognition on Scanning Probe Microscopy Images Using Computer Vision and Deep Learning
Source: Nanomaterials (Basel). 2020 Jun 30;10(7):1285. doi: 10.3390/nano10071285 (PMC7408120; doi:10.3390/nano10071285)
Supplement: Supplementary file 1 [file nanomaterials-10-01285-s001.pdf]

nanomaterials

Nanoparticle Recognition on Scanning Probe Microscopy Images Using Computer Vision and Deep Learning

Alexey G. Okunev [1,2], Mikhail Yu. Mashukov [3], Anna V. Nartova [2,3] and Andrey V. Matveev [2,3,*]

1 Novosibirsk State University Higher College of Informatics, Russkaja Str. 35, 630058 Novosibirsk, Russia; okunev73@mail.ru
2 Boreskov Institute of Catalysis SB RAS, pr. Acad. Lavrentieva, 5, 630090 Novosibirsk, Russia; avnartova@gmail.com
3 Scientific-Educational Center "Machine Learning and Big Data Analysis", Novosibirsk State University, Pirogova Str. 1, 630090 Novosibirsk, Russia; mashukovm@mail.ru
* Correspondence: matveev.nsu@mail.ru; Tel.: +007-383-363-4390

1. Comparison with WSxM Software

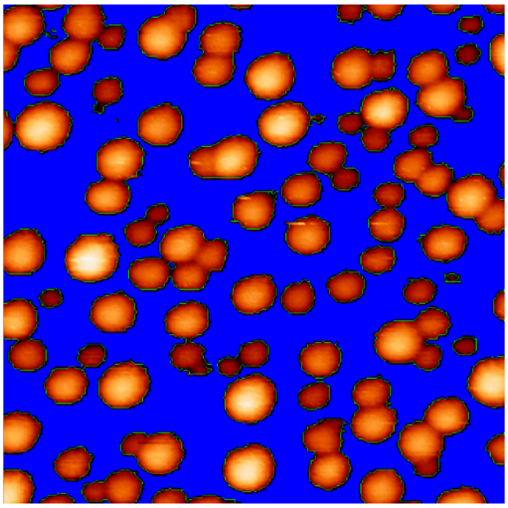

(a)

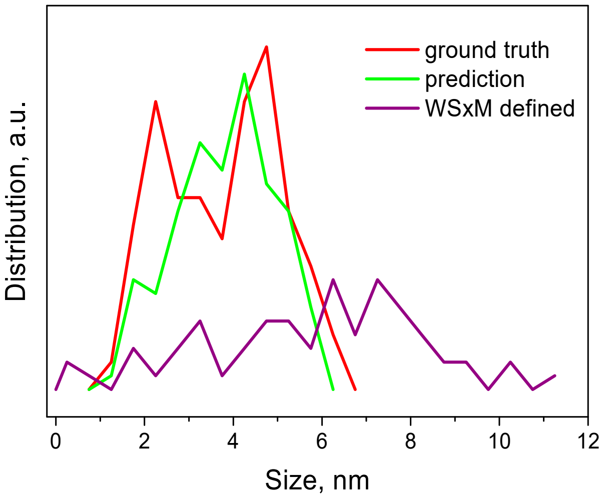

(b)

Figure S1. Processing of Image 2: (**a**) results of the application of the "flooding" procedure in WSxM software products; (**b**) size distributions of the 69 contours defined by WSxM software (purple), 118 particles recognized in our work (green) as well as 146 contours of ground truth (red).

Table S1. Processing of Image 2: number of particles and their mean particle sizes calculated from histograms of different types of the contours.

Method for determining particle size	Number of particles	Mean particle size, nm	Standard error of mean
Procedure "flooding", WSxM software	69 [1]	5.78 [1]	0.29 [1]
Neural network Cascade Mask-RCNN, used in this work (predicted)	118	3.82	0.1
Ground truth	146	3.75	0.11

[1] Depends on threshold.

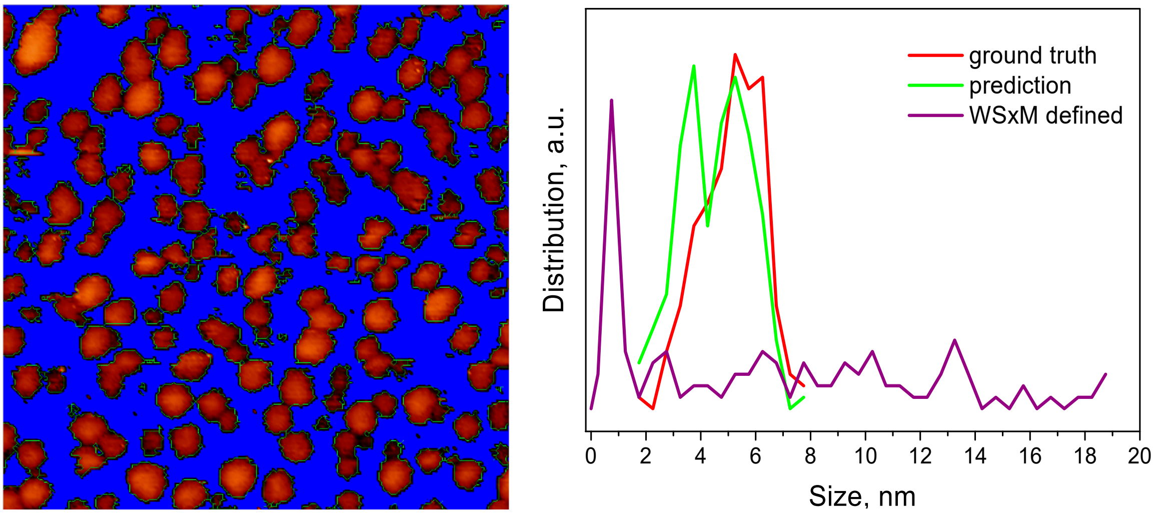

(**a**) (**b**)

Figure S2. Processing of Image 3: (**a**) results of the application of the "flooding" procedure in WSxM software products; (**b**) size distributions of the 115 contours defined by WSxM software (purple), 190 particles recognized in our work (green) as well as 172 contours of ground truth (red).

Table S2. Processing of Image 3: number of particles and their mean particle sizes calculated from histograms of different types of the contours.

Method for determining particle size	Number of particles	Mean particle size, nm	Standard error of mean
Procedure "flooding", WSxM software	115 [1]	6.46 [1]	0.50 [1]
Neural network Cascade Mask-RCNN, used in this work (predicted)	190	4.52	0.09
Ground truth	172	5.13	0.09

[1] Depends on threshold.

2. Web Service

To make it possible for any user, anywhere in the world to use the results of our work on particle recognition on STM images, we have been developing a web service "ParticlesNN"(http://particlesnn.nsu.ru/).

In the main menu, the user can upload microscopic image in BMP or TXT format. In the second case, the files additionally contain the scale and intensity of the STM-signal. Then, the user goes to a subdirectory with the name of the file and starts data processing, which takes about one minute. The output shows a grey-scale BMP image with the contours of nanoparticles found by the neural network, as well as contours, fitted by Gaussian postprocessing. The results of simple statistical processing are also available on the page:

1. Number of particles N;
2. The area of the image covered by the recognized articles *s*;
3. Midsurface diameter d_{ms}, calculated as $(4 \times s/\pi)^{1/2}$ and its standard deviation;
4. Average projected area diameter d_{av} and its standard deviation;
5. Density of the particles.

If the uploaded file was initially in TXT format containing the scale of the STM-picture, then all the characteristics will be in measuring units used in the microscope acquisition program. Otherwise, they are in pixels.

The page also displays a distribution diagram of the average projected area diameters d_{av} of the particles. For a more detailed statistical analysis, we recommend using more specialized programs.

Produced image files and statistics in CSV format can be downloaded from the page. Images with the contours of found nanoparticles are presented in BMP and JSON formats in two variants: contours of nanoparticles initially predicted by the network and the ones fitted by Gaussian postprocessing. It is very useful and important for a user, that the JSON file can be edited in the LabelMe program (https://github.com/wkentaro/labelme) and uploaded again to the main page. This lets the user correct the contours found by the neural network, for example, to remove mistakenly defined contours or add the missed particles. Our service results are not in the immutable truth figures, but are open for adjustments in the results obtained and postprocessing them.

The statistical results are presented in a CSV (comma-separated values) format which contains coordinates of the particles, their areas, and average projected area diameters d_{av} for both particles found by the neural network and particles fitted by Gaussian postprocessing.

There is one more useful function. On the main page, the user can also mark a set of necessary files and get the joined statistical information for all particles in the chosen files.
